# Maximal Fat Oxidation During Exercise Is Already Impaired in Pre-pubescent Children With Type 1 Diabetes Mellitus

**DOI:** 10.3389/fphys.2021.664211

**Published:** 2021-04-09

**Authors:** Solenne Fel, Emmanuelle Rochette, Guillaume Walther, Stéphane Echaubard, Bruno Pereira, Etienne Merlin, Daniel Terral, Pascale Duché

**Affiliations:** ^1^Pédiatrie Générale et Multidisciplinaire, CHU Clermont-Ferrand, Clermont-Ferrand, France; ^2^INSERM, CIC 1405, Unité CRECHE, Université Clermont Auvergne, Clermont-Ferrand, France; ^3^Laboratoire IAPS, Université de Toulon, Toulon, France; ^4^LaPEC, Avignon Université, Avignon, France; ^5^Délégation de la Recherche Clinique et Innovations, CHU Clermont-Ferrand, Clermont-Ferrand, France; ^6^INRA, UMR 1019 UNH, ECREIN, Université Clermont Auvergne, Clermont-Ferrand, France

**Keywords:** glycemia, glucose metabolism, physical activity, pediatric, metabolic

## Abstract

**Objective:** We evaluated substrate utilization during submaximal exercise, together with glycemic responses and hormonal counter-regulation to exercise, in children with type 1 diabetes mellitus (T1DM).

**Methods:** Twelve pre-pubescent children with T1DM and 12 healthy children were matched by sex and age. Participants completed a submaximal incremental exercise test to determine their fat and carbohydrate oxidation rates by indirect calorimetry. Levels of glycemia, glucagon, cortisol, growth hormone, noradrenaline, adrenaline, and insulin were monitored until 120 min post-exercise.

**Results:** Absolute peak oxygen uptake (VO_2_ peak) was significantly lower in the children with T1DM than in the healthy controls (1131.4 ± 102.5 vs. 1383.0 ± 316.6 ml.min^−1^, *p* = 0.03). Overall carbohydrate and lipid oxidation rates were the same in the two groups, but for exercise intensities, higher than 50% of VO_2_ peak, fat oxidation rate was significantly lower in the children with T1DM. The absolute maximal lipid oxidation rate was significantly lower in the T1DM children (158.1 ± 31.6 vs. 205.4 ± 42.1 mg.min^−1^, *p* = 0.005), and they reached a significantly lower exercise power than the healthy controls (26.4 ± 1.2 vs. 35.4 ± 3.3 W, *p* = 0.03). Blood glucose responses to exercise were negatively correlated with pre-exercise blood glucose concentrations (*r* = −0.67; *p* = 0.03).

**Conclusion:** Metabolic and hormonal responses during sub-maximal exercise are impaired in young children with T1DM.

## Introduction

Type 1 diabetes mellitus (T1DM) is an autoimmune metabolic diseases associated with deficient insulin secretion and resulting in impaired carbohydrate metabolism (dysglycemia; Galassetti and Riddell, 2013). The incidence of T1DM in children has increased worldwide by 3–4% per year in recent decades. The age of onset T1DM has also decreased in recent decades in Europe (Tuomilehto et al., 2020). The most substantial increases in T1DM incidence have been reported in children younger than 5 years of age (Atkinson et al., 2014).

Although regular exercise increases cardiorespiratory fitness, improves insulin function, and reduces risk of diabetes-related complications, children with T1DM tend to be less physically active than their healthy peers (Valerio et al., 2007) and so can be less physically fit (e.g., lower VO_2max_ and muscle strength; Lukács et al., 2012; Galassetti and Riddell, 2013). This lower physical activity in children with T1DM may be explained by difficulties in managing insulin administration during exercise to avoid unexpected hypo- and/or hyperglycemic episodes during and after physical activity. However, improved treatment regimens (pumps, or long-lasting background insulins) and glycemic management (continuous glucose monitoring and intermittently scanned continuous glucose monitoring systems), to easily measure glucose and manage adaptations around exercise, are a major advance for a more active lifestyle (Moser et al., 2020).

As insulin is not endogenously regulated, insulin enters the system from the injection site, and not *via* the portal vein with first pass by the liver, and so peripheral tissue experiences relative hyperinsulinemia. During submaximal exercise, the subcutaneously injected insulin resorption toward the bloodstream is accelerated, resulting in an insulin boost that increases glucose uptake by muscle and suppresses endogenous glucose production (Riddell et al., 2017). Furthermore, the counter-regulatory hormones released in response to exercise [glucagon, epinephrine, norepinephrine, growth hormone (GH), and cortisol] are disturbed in cases of complication of longstanding or poorly controlled diabetes (Galassetti and Riddell, 2013). This may lead to a higher risk of hypoglycemia during or after exercise.

In T1DM, the muscles’ ability to effectively adapt to the increased energy substrate requirements of exercise is impaired (Galassetti and Riddell, 2013). Comparing adults with T1DM with those considered healthy, it seems that the consumption of lipids and carbohydrates is different during exercises with an intensity <50% VO_2max_ (Raguso et al., 1995; Robitaille et al., 2007; Dumke et al., 2013). In a study assessing adolescents and young adults, there was no significant difference in the oxidation of substrates for exercise at 60% VO_2max_ between patients with T1DM and controls (Riddell et al., 2000).

There is little data detailing energy metabolism, hormonal counter-regulation, and glycemic response to exercise in children with T1DM and even less in pre-pubescent children. It was hypothesized that the muscle impairment in T1DM occurs in the very early stages of life, so that even young children with T1DM may have impaired physiological adaptations to exercise. The purpose of this study was to compare, between pre-pubescent children with T1DM and healthy controls, substrate oxidation rates during sub-maximal exercise and evaluate the glycemic response and hormonal counter-regulation to exercise.

## Materials and Methods

### Participants

Twelve pre-pubescent (Tanner stage I) children diagnosed with T1DM for more than 1 year and with no evidence of T1DM-related tissue complications were compared to an equal number of healthy controls recruited as peers and matched for age and sex. Sexual maturity was assessed by medical doctors using the indices of pubic hair and male genital or female breast development as described by Tanner. All the subjects were monitored at the pediatric unit of the Clermont-Ferrand University Hospital, France.

Participants were excluded if they had a physician-diagnosed infection, thyroid disorders, were unable to perform the prescribed exercise, and/or had a contraindication to physical exercise (cardiorespiratory pathologies incompatible with submaximal exercise and orthopedic or neurologic dysfunctions that ruled out pedaling). Their physical activity level (PAL) was determined according to the French questionnaire on physical activity practiced (QAPE-week; Tessier et al., 2007). Physical activity level (for involvement in physical activities at school, physical activities outside school, and physical exercise and sport) was scored from 0 to 48, sedentarity (time spent in front of a screen) was scored from 0 to 28, and intensity of physical activity was scored from 0 to 7.

This study was approved and conducted in accordance with the recommendations of the Comité de Protection des Personnes (CPP) Est II, (no. 2016-A01837-44). All the participants and their parents provided written informed consent in accordance with the Declaration of Helsinki.

### Experimental Procedure

**Figure 1:**
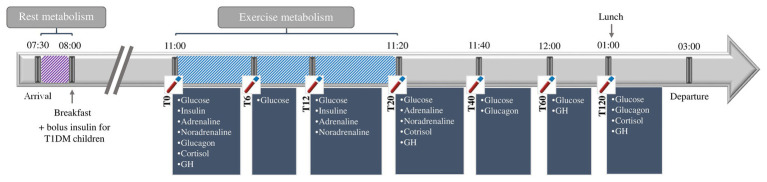
Study design.

Prior to the day of evaluation, all the participants were asked to consume a prescribed diet in accordance with dietary reference intakes for sex and age [Institute of Medicine (US) Committee to Review Dietary Reference Intakes for Vitamin D and Calcium et al., 2011], with a standardized quantity of proteins, lipids, and carbohydrates. Recommended nutritional intakes according to age were 1,600 kcal/day for children aged 6–7 years, 1,800 kcal/day for 8–9 years, and 2,000 kcal/day for 10–12 years, with standardized proportions of carbohydrates (52%), fat (35%), and protein (13%). For children with T1DM, the basal and bolus insulin doses were unchanged on the day prior to the evaluation (20% of the total daily insulin dose defined by 1 unit/kg of body weight. Children refrained from strenuous physical activity for at least 24 h beforehand. Participants arrived fasted and attended the hospital laboratory from 07:30 am to 03:00 pm (Figure 1). Resting metabolic rate was recorded for 10 min after the participant had sat quietly for 20 min, in accordance with a previous published protocol (Compher et al., 2005). Identical breakfasts and lunches, standardized and in accordance with the participants’ dietary reference intake, were given, respectively, at 08:00 am and 01:00 pm. For children with T1DM, the usual insulin bolus dose was administrated before breakfast (around 08:00 am).

At 11:00 am (at least 2 h after bolus insulin), the participants completed a graded exercise test, to the point of volitional fatigue, on an electromagnetically braked cycle ergometer with continuous gas collection and heart rate monitoring. Children were familiarized with the cycle ergometer, laboratory environment, and exercise protocols. The pedal cranks and seat height were adjusted for each child. To avoid circadian variance, experiments were always performed at the same time in the morning. Measurements were performed in quiet, gently lit rooms, at an ambient temperature of 23°C and relative humidity 50–65%. Following a 2-min warm-up consisting of unloaded pedaling, the participants started cycling at 10 W. Their work rate was increased stepwise by 10 W every 3 min thereafter. If the participant’s heart rate was unstable (heart rate variation more than ±5 beats per minute) at any given stage, this stage was extended for up to 5 min to obtain a heart rate stable within ±5 beats. When the respiratory exchange ratio (RER) was greater than or equal to 1.00, indicating the absence of fat oxidation (Riddell et al., 2008), the participant’s work rate was increased by the same increments, but at 1-min intervals until volitional fatigue was reached. This modified protocol was to profile both substrate oxidation, and to determine VO_2_ peak (Riddell et al., 2008).

All the exercise tests were performed on a Cyclus 2 ergometer (RBM Elektronik-Automation GmbH, Leipzig, Germany). O_2_ consumption (VO_2_) and CO_2_ production (VCO_2_) were measured breath by breath through a mask connected to an O_2_ and CO_2_ analyzer (MetaMax 3b, Cortex Biophysik, Leipzig, Germany).

Ventilatory parameters were averaged every minute during the submaximal exercise test and during the 10-min recovery period. Heart rate was monitored continuously throughout the duration of the tests (Polar RS800cx monitor, Polar, Finland).

### Data Analysis

Indirect calorimetry is the recognized standard method for quantifying substrate oxidation rates at rest and during exercise (Frayn, 1983). The VO_2_ and VCO_2_ values were averaged over the last minute of each work rate, with the results then being used to calculate fat and carbohydrate oxidation over a wide range of exercise intensities for each participant (Achten et al., 2002) using Péronnet and Massicotte’s equation: lipids (mg.min^−1^) = 1.6946 × VO_2_ − 1.7012 × VCO_2_; carbohydrate (CHO; mg.min^−1^) = 4.585 VCO_2_ − 3.2255 VO_2_ (Péronnet and Massicotte, 1991).

For each individual, a best-fit polynomial curve was constructed for fat and CHO oxidation rate (expressed as mg.min^−1^) vs. exercise intensity (expressed as a percentage of the VO_2_ peak). Each individual curve was then used to determine the peak fat oxidation rate and the exercise intensity associated with the maximal fat oxidation (MFO) rate (Achten et al., 2002).

Predictive VO_2_ peak (ml.min^−1^) was calculated with the linear regression equations (Cooper et al., 1984). For boys, *y* = 52.8 × body weight (kg) − 303.4 and for girls, *y* = 28.5 × body weight (kg) + 288.2. Mean theoretical predicative VO_2_ peak was 1105.5 ± 71.3 ml.min^−1^ for children with T1DM and 1131.0 ± 110.9 ml.min^−1^ for the healthy controls (*p* > 0.05).

### Blood Samples and Hormonal Assessment

Venous blood samples were taken *via* an indwelling catheter at 11:00 (T0), 11:06 (T6), 11:12 (T12), 11:20 (T20), 11:40 (T40) am and 12:00 (T60), 01:00 (T120) pm, into EDTA K2 Vacutainer® tubes (Becton Dickinson, Franklin Lakes, NJ, United States) were used for the analysis of adrenaline and noradrenaline, in Vacutainer® Lithium Heparin tubes (Becton Dickinson, Franklin Lakes, NJ, United States) for analysis of glycemia, in EDTA K3/Aprotinin Vacutainer® tubes (Becton Dickinson, Franklin Lakes, NJ, United States) for the analysis of glucagon, and in Vacutainer® SST II Advance tubes (Becton Dickinson, Franklin Lakes, NJ, United States) for the analysis of cortisol, insulin, and GH. Samples collected were analyzed immediately. Plasma adrenaline and noradrenaline levels were measured by high performance liquid chromatography. Serum GH levels were measured using chemiluminescent immunometric assay (Immulite 2000, Siemens Healthcare Diagnostics; analytical sensitivity = 0.1 ng/ml). Serum cortisol levels were measured by chemiluminescence on an ADVIA Centaur XP Analyzer (Siemens Healthcare Diagnostics; analytical sensitivity = 27.6 nmol/L). Plasma glycemia levels were measured using hexokinase, UV/bichromatic end point (Dimension Vista® 1500, Siemens Healthcare Diagnostics; analytical sensitivity = 0.06 mmol/L). Plasma glucagon levels were measured using radioimmunoassay (Glucagon EURIA, EuroDiagnostica, Malmo, Sweden; analytical sensitivity 10 ng/L). Serum insulin levels were measured using chemiluminescent immunometric assay (Immulite 2000, Siemens Healthcare Diagnostics; analytical sensitivity = 2 μIU/ml).

### Statistics

Sample size was estimated according to (i) the CONSORT 2010 statement, extension to randomized pilot and feasibility trials (Eldridge et al., 2016) and (ii) Cohen’s recommendations (Cohen, 1988), which define effect size bounds as small (ES: 0.2), medium (ES: 0.5), and large (ES: 0.8, “grossly perceptible and therefore large”). More precisely, with 12 subjects per group (pre-pubescent children and healthy children), an effect size greater than 1.2 can be highlighted for a two-sided type I error of 5% and a statistical power of at least 80%.

Continuous data were expressed, according to the statistical distribution, as mean and SD or as median (interquartile range). The assumption of normality was assessed with the Shapiro-Wilk test.

The comparisons between groups (i.e., children with T1DM vs. healthy controls) concerning the non-repeated quantitative parameters (age, HbA1c, time from diagnosis, body mass, BMI, absolute VO_2_ peak, rest metabolism, heart rate, and physical activity score) were performed using the Student *t*-test or the Mann-Whitney test when assumptions required for the *t*-test were not met. Homoscedasticity was analyzed using the Fisher-Snedecor test. The relationship between the delta between the end of exercise and pre-exercise value for glucose concentrations was explored using the Spearman correlation coefficient.

To account for the between- and within-participant variability caused by several measures being made for the same subject, random-effects models for the correlated data (insulin, adrenalin, noradrenalin, GH, cortisol, glucagon, and glycemia) were then executed. Owing to unverified assumption of independence, these models were preferred over the usual statistical tests, which are inappropriate. Time-point evaluations, group of participants (T1DM vs. healthy controls), and their interactions were considered as fixed effects. Subject was considered a random effect (slope and intercept). The normality of the residuals from these models was studied as stated above, using the Shapiro-Wilk test. When appropriate, the data were log-transformed to achieve normality of the dependent endpoint. A Sidak *post hoc* test was applied to correct the type-I error due to multiple comparisons. Random-effects models were also performed to study the relationships between percentage levels of %VO_2_ peak and CHO oxidation, fat oxidation, power, and heart rate.

The statistical analyses were performed using Stata software version 15 (StataCorp, College Station, United States). Tests were two-sided with the type-I error set at 5%. Special attention was also paid to the magnitude of differences and to clinical relevance. The results are expressed with Hedges’ effect sizes and 95% confidence intervals.

## Results

### Participant’s Characteristics

All the children who were enrolled completed the exercise tests without adverse effects (such as a cardiovascular event, lightheadedness, or any general malaise). The participants’ characteristics are summarized in Table 1. For children with T1DM, the strategy for insulin administration was a continuous subcutaneous insulin pump infusion for three of the participants and multiple daily insulin injections for nine. Their total insulin dose was 0.77 IU.kg^−1^ day^−1^ (range 0.53–1). There was no significant difference between the children with T1DM and the healthy children in resting metabolism, relative VO_2_ peak, BMI-for-age percentile, rest heart rate, maximal heart rate, or physical activity level score (Table 1). Absolute VO_2_ peak was significantly lower in the children with T1DM than in the control group (1131.4 ± 102.5 vs. 1383.0 ± 316.6 ml.min^−1^, respectively, *p* = 0.03).

**Table 1:** Participants’ characteristics.

	T1DM (*n* = 12)	Healthy controls (*n* = 12)	*p*	Hedge’s *g* (95% CI)
Sex (male/female)	5/7	5/7		
Age (years)	9.3 ± 1.4	9.3 ± 1.5	0.97	−0.01 (−0.81; 0.19)
HbA1c (%)	7.6 ± 0.9	NA		
Time from diagnosis (years)	4.0 ± 2.7	NA		
Body mass (kg)	28.6 ± 3.6	31.0 ± 8.7	0.38	0.35 (−0.46; 1.16)
BMI (kg/m^2^)	16.0 ± 1.2	17.0 ± 2.5	0.20	**0.52 (−0.30; 1.33)**
BMI-for-age percentile^a^	41.4 ± 23.4	50.8 ± 27.1	0.37	0.36 (−0.45; 1.16)
Absolute VO_2_ peak (ml/min)	1131.4 ± 102.5	1383.0 ± 316.6	**0.03**	**1.03 (0.19; 1.85)**
Relative VO_2_ peak (ml/kg/min)	39.8 ± 3.6	44.7 ± 9.7	0.12	**0.65 (−0.15; 1.43)**
Rest metabolism (kcal/day)	1349.2 ± 296.0	1291.9 ± 335.9	0.81	−0.17 (−0.95; 0.60)
Rest heart rate (beat/min)	90 ± 12	87 ± 5	0.67	−0.36 (−1.25; 0.52)
Maximal heart rate (beat/min)	173 ± 20	183 ± 13	0.27	**0.57 (−0.37; 1.51)**
Physical activity score	12.8 ± 4.2	16.0 ± 5.4	0.14	**0.64 (−0.18; 1.46)**
Sedentary score	6.5 ± 3.5	7.9 ± 2.8	0.36	0.42 (−0.44; 1.29)
Glycemia pre-exercise T0 (g.L^−1^)	2.14 ± 0.51	0.98 ± 0.14	**<0.001**	**−3.03 (−4.43; −1.64)**
Glycemia post-exercise T20 (g.L^−1^)	1.69 ± 0.57	1.01 ± 0.16	**0.04**	**−1.59 (−2.68; −0.50)**

Data are means ± SD; T1DM, type 1 diabetes mellitus; BMI, body mass index; NA, not applicable; CI, confidence interval; SD, standard deviation.

a Each individual BMI was plotted on the American Academy of Pediatrics BMI for-age growth charts to obtain a percentile ranking. Hedge’s *g* is a measure of effect size defined as small (ES: 0.2), medium (ES: 0.5), and large (ES: 0.8). Values in bold are statistical significant at *p* < 0.05 or with medium to large effect size.

### Oxidation of Metabolites

**Figure 2:**
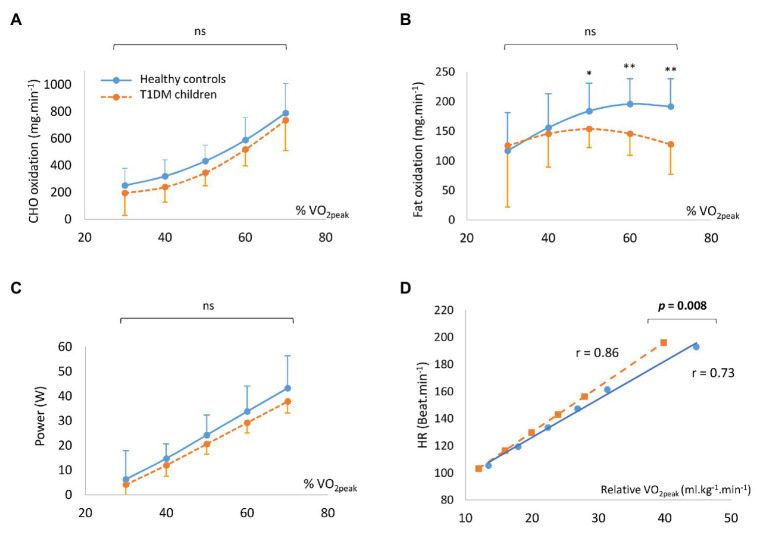
The oxidation rates of carbohydrates **(A)** and fat **(B)**, in conjunction with power output **(C)** according to the percentage of VO_2_ peak. Individual regression for heart rate (HR) vs. the corresponding relative VO_2_ peak for children with type 1 diabetes mellitus (T1DM) and healthy controls **(D)**. Data are means ± SD.

The oxidation rates of lipids and carbohydrates as a function of percentage of VO_2_ peak are presented in Figure 2. Global carbohydrate and lipid oxidation rate were the same in the two groups, but for exercise intensities, higher than 50% up to 70% of VO_2_ peak, fat oxidation rate was significantly reduced in the children with T1DM (Figures 2A,B). Values of power were the same for the two groups, regardless of the intensity of exercise (Figure 2C). Heart, rate vs. relative VO_2_ peak during exercise was significantly displaced upward and displayed a greater slope comparing children with T1DM and healthy controls (Figure 2D).

### Maximal Fat Oxidation

**Figure 3:**
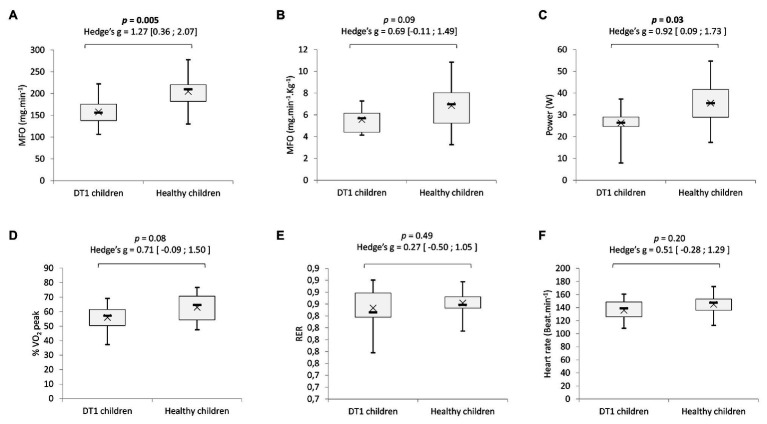
Comparison of absolute **(A)** and relative **(B)** maximal fat oxidation rate (MFO). Power **(C)**, percentage of VO_2_ peak **(D)**, respiratory exchange ratio **(E)**, and heart rate **(F)** at the MFO. Boxes represent interquartile ranges. Whiskers give minimum and maximum values. Data are mean (x) and median (−).

For children with T1DM and healthy children, the respective absolute MFO was 158.1 ± 31.6 vs. 205.4 ± 42.1 mg.min^−1^ (*p* = 0.005) and relative MFO was 5.6 ± 1.2 vs. 6.9 ± 2.2 mg.min^−1^.kg^−1^ [*p* = 0.09, Hedge’s *g* = 0.70 (−0.10; 1.50); Figures 3A,B].

Power, percentage of VO_2_ peak, RER, and heart rate (HR) of exercise to achieve MFO are shown in Figures 3C–F. Between the children with T1DM and their healthy peers, there was no difference in percentage of VO_2_ peak [56.0 ± 9.3 and 63.0 ± 9.6%, *p* = 0.08, Hedge’s *g* = 0.71 (−0.09; 1.50); Figure 3D], in the RER [0.85 ± 0.03 vs. 0.86 ± 0.02, *p* = 0.49, Hedge’s *g* = 0.27 (−0.50; 1.05); Figure 3E] or the heart rate [136 ± 17 and 145 ± 17 beats.min^−1^, *p* = 0.20, Hedge’s *g* = 0.51 (−0.28; 1.29); Figure 3F] or the at the point of MFO. However, children with T1DM reached MFO at a significantly lower exercise power than healthy controls (26.4 ± 1.2 and 35.4 ± 3.3 W, *p* = 0.03; Figure 3C).

### Hormonal Counter-Regulation and Glycemic Response to Exercise

**Figure 4:**
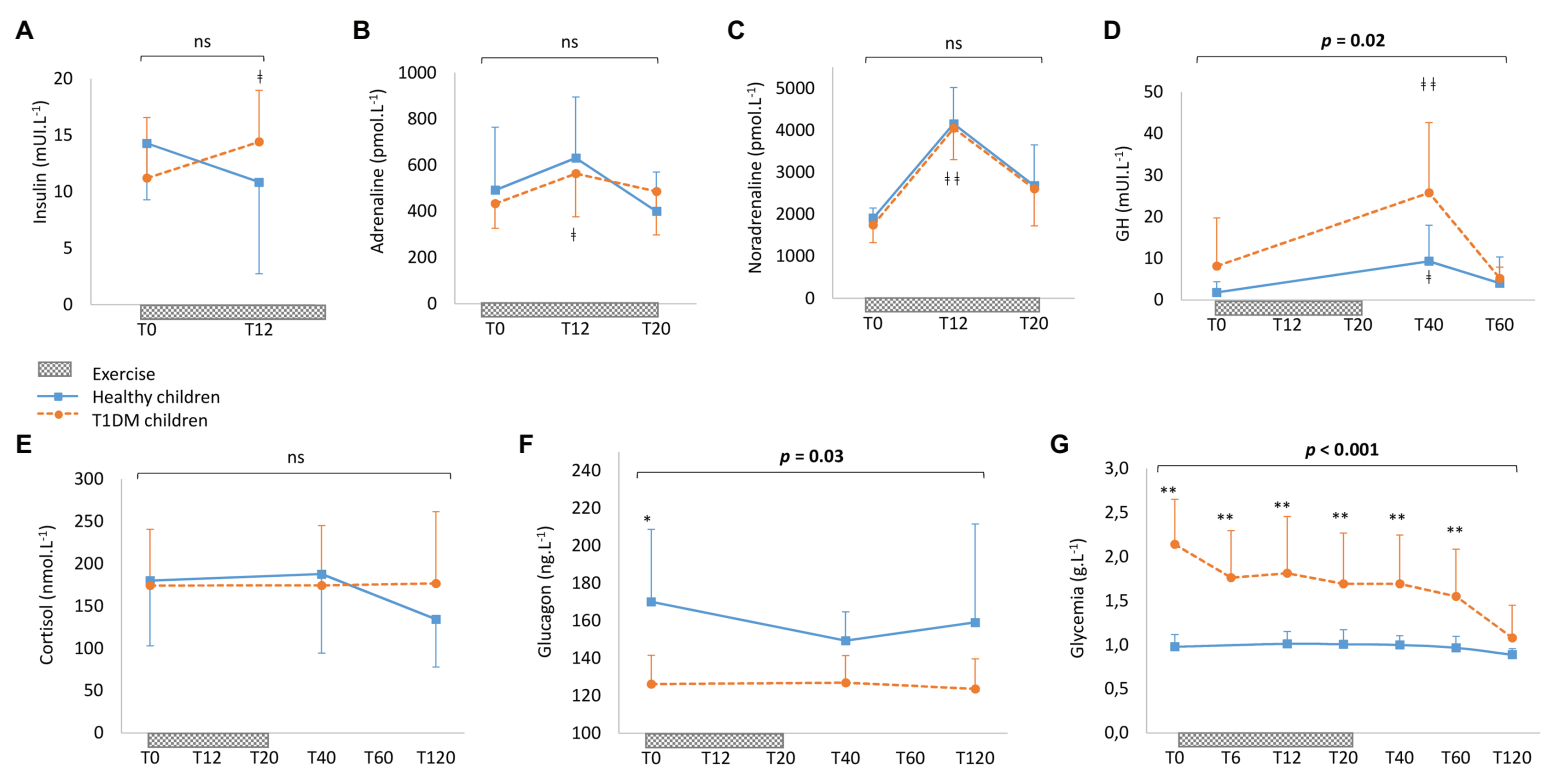
Blood concentrations of insulin **(A)**, adrenaline **(B)**, noradrenaline **(C)**, growth hormone **(D)**, cortisol **(E)**, glucagon **(F)**, and glycemia **(G)** in healthy controls (solid line) and children with T1DM (dotted line). Incremental exercise starts at T0 and finishes at T20. Data are means ± SD. ^∗^Significantly different between healthy controls and T1DM children at *p* < 0.05. ^∗∗^Significantly different at *p* < 0.001. ^ǂ^Significantly different from T0 at *p* < 0.05. ^ǂ ǂ^Significantly different from T0 at *p* < 0.001.

**Figure 5:**
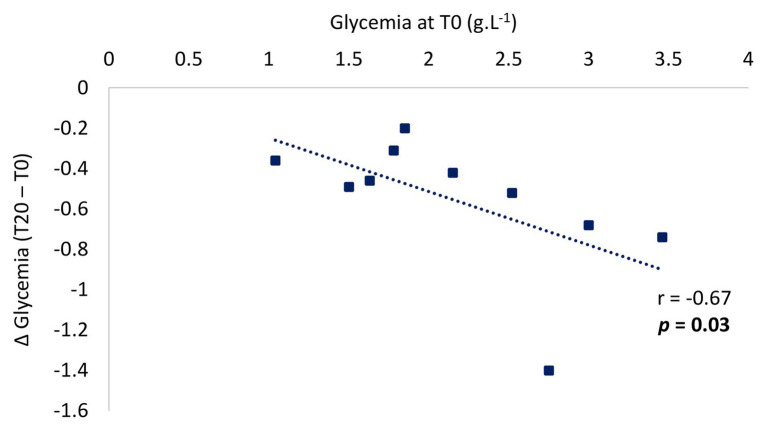
Individual pre-exercise glycemia values according to the drop in glycemia during post-exercise in children with T1DM. The change in glucose concentrations during exercise was calculated from the delta of the end of exercise (T20) and pre-exercise value (T0). Two of the blood glucose data at T20 are missing due to an analytical problem.

The variation in the concentrations of hormones in the blood in response to exercise are presented in Figure 4. There was no difference between the two groups in overall levels of insulin, adrenaline, noradrenaline, and cortisol in response to exercise (Figures 4A–C,E). At T12, compared to pre-exercise (T0), adrenaline and noradrenaline increased significantly in both groups. However, insulin concentrations increased only in the group of children with T1DM. Overall, the concentration of glucagon was significantly lower in the children with T1DM (*p* = 0.03, Figure 4F) and stable after exercise in both groups. While the level of GH increased significantly at T40 in both groups, it was higher in children with T1DM overall (*p* = 0.02, Figure 4D). Finally, glycemia was significantly higher during exercise in children with T1DM (*p* < 0.001) and decreased significantly compared to pre-exercise level (2.14 ± 0.55 g.L^−1^ in pre-exercise vs. 1.08 ± 0.41 g.L^−1^ at T120 min, *p* < 0.001, Figure 4G). In the children with T1DM, the blood glucose response to exercise was negatively correlated with the pre-exercise blood glucose level (*r* = −0.67; *p* = 0.03; Figure 5, two of the blood glucose data at T20 are missing due to an analytical problem).

## Discussion

The first main finding from the present study was that during submaximal exercise, the oxidation rates of lipid and carbohydrates were lower in pre-pubescent children with T1DM than in healthy controls, with a significantly lower maximal fat oxidation rate. Secondly, in children with T1DM, the change in glucose levels in response to exercise was negatively correlated with the pre-exercise glucose concentration. Finally, hormonal counter-regulation (for GH, glucagon, and insulin) to exercise differed in those with T1DM. These results suggest a T1DM-related dysregulation in the metabolic and hormonal response to exercise even in young children with T1DM who have adequate glycemic control (HbA1c = 7.6%).

Interestingly, there were lower carbohydrate and fat oxidation rates in the pre-pubescent children with T1DM compared to the healthy children. A previous study had shown that during exercise, in adults with T1DM, the rate of lipid oxidation was lower in hyperglycemia than in euglycemia (Jenni et al., 2008). Lower fat oxidation in patients with diabetes may be associated with an inability to decrease circulating insulin levels, inhibiting lipolysis and leading to a possible increase in the hepatic uptake of fatty acids, together with an increase in GLUT4 recruitment to the cellular membrane and activation of muscle hexokinase, enabling the phosphorylation of glucose and increasing glucose muscle uptake (de Jesus et al., 2019). Additionally, there were a significantly lower MFO rate and power at MFO (Figures 2, 3) in children with T1DM. The ability to oxidize lipids and carbohydrates during incremental exercise reflects a profile of metabolic fitness correlated to the physiological status of the muscles (Brun et al., 2012; Zakrzewski and Tolfrey, 2012). There could, therefore, be an impaired muscle function during exercise, especially since it has been demonstrated that in young adults with T1DM, skeletal muscle mitochondria have irregularly organized cristae at the ultrastructural level, a decreased ability to produce adenosine triphosphate in response to increasing energy demand, and an increase in complex III-derived reactive oxygen species subsequent to fatty acid oxidation (Monaco et al., 2020). Furthermore, research has found reductions in skeletal muscle mitochondrial oxidative phosphorylation in adolescents with T1DM, which may be linked to insulin resistance, potentially through altered redox signaling (Fisher-Wellman and Neufer, 2012; Monaco et al., 2018). Finally, in poorly controlled diabetes, reduction in muscle fiber size and changes in the distribution of muscle fiber types have been observed, with an increase in the percentage of glycolytic/fast-twitch (type II) muscle fibers and an impairment in oxidative capacities in adults with T1DM (Fritzsche et al., 2008; Galassetti and Riddell, 2013). All these observations suggest that even in young pre-pubertal children, there may be distorted muscular function and architecture, reflected by an impaired energy metabolism during exercise.

One determinant of the capacity of an individual to adequately adapt to exercise is cardiorespiratory fitness. Although the validated protocol used to profile both substrate oxidation, and to determine VO_2_ peak (Riddell et al., 2008), may underestimate the true aerobic capacity (i.e., VO_2max_) of the subjects, all children enrolled completed exercise test until reach the VO_2_ peak without adverse effect (such as a cardiovascular event, lightheadedness, or general malaise), and for children with T1DM without exercise-induced hypoglycemia. In this context, there was a significantly lower VO_2_ peak in the children with T1DM in this study, similar to the findings of previous studies (Lukács et al., 2012; Galassetti and Riddell, 2013). Consequently, since VO_2_ peak is an indication of cardiorespiratory fitness, and since exercise has a positive longitudinal influence on physical fitness, pre-pubescent children with T1DM might simply be less physically active. Similar physical fitness was reported (same VO_2max_, heart rate during exercise or lactate threshold) between controls and non-sedentary adolescents with T1DM (Nascimento et al., 2017), suggesting that in our results, the lower VO_2_ peak could be due to a lower level of physical activity. When physical activity levels were evaluated by a questionnaire, we found no significant difference in the levels of physical activity between our two groups. However, when the physical activity score was considered, there was a large effect size, suggesting that children with T1DM in this study could be less physically active overall than the healthy controls (particularly with lower physical intensity score and number of physical activities per day) as already reported in previous studies in adolescents and young adults with T1DM (Valerio et al., 2007; Krause et al., 2011).

In the present study, heart rate according to the relative VO_2_ peak (Figure 2D) during exercise was significantly higher in children with T1DM, suggesting impaired cardiac adaptation to exercise, which may contribute to poorer exercise capacity. In another study, decreased physical fitness evaluated by a 6-min walking test showed that adolescents with T1DM were less fit than controls, independently of HbA1c level (>8%; Jegdic et al., 2013). An elevation of resting heart rate and a reduction of maximum heart rate, resulting in a lower heart rate response to exercise, are common manifestations of cardiac autonomic dysfunction and/or neuropathy that are manifestations of uncomplicated T1DM, even in the preteen years of life (Krause et al., 2011; Wilson et al., 2017). In our study, although not statistically significant, children with diabetes had a higher rest heart rate and lower maximal heart rate than the controls, suggesting an early autonomic cardiac disturbance.

This lower aerobic capacity in children with T1DM may be related to early defects in the delivery of blood *via* the microcirculation to the skeletal muscle. Additionally, it may contribute to impairments in complex IV capacity in the mitochondrial respiratory chain (Heyman et al., 2020). Capacity to transport and utilize oxygen affects skeletal muscle function and exercise capacities (Lanfranconi et al., 2014). A reduction in VO_2_ peak (considered as an individual index of aerobic fitness) is evidence of the cardiovascular system’s inability to deliver O_2_ to the skeletal muscle. However, during exercise, an impairment in the utilization of O_2_ at the tissue could also be involved in a reduction of exercise capacities. Previous research had found that patients with T1DM displayed blunted perfusion and oxygen extraction in active skeletal muscle at moderate-to-maximal exercise intensities (Tagougui et al., 2015; Heyman et al., 2020). Accordingly, a greater increase in heart rate, along with an increase in the percentage of relative VO_2_ peak in children with T1DM compared to the healthy control found in this study, could be a compensatory adaptation to increase O_2_ delivery to the exercising muscle.

Moderate-intensity exercise leads to a marked increase in glucose uptake. However, in those with T1DM who are maintaining insulin levels, there is mismatch of glucose production to uptake during exercise resulting from an inadequate hepatic response (Camacho et al., 2005). Hence the major risk in patients with T1DM is post-exercise hypoglycemia. Like in other studies (Galassetti and Riddell, 2013; Ben Brahim et al., 2015; Riddell et al., 2019), a negative correlation between the pre-exercise glucose concentration and the drop in glucose during exercise in pre-pubescent children with T1DM was observed here. However, with a median pre-exercise glucose level of 214 mg/dl there was no post-exercise hypoglycemic episode.

Given that the hormonal response does not differ between adolescents and pre-pubescents with diabetes (Adolfsson et al., 2012), it seems that hormonal responses to exercise are not affected by pubertal stage in T1DM. Pre-pubescent children with T1DM maintained adequate hormonal response to exercise for catecholamines, and cortisol presented similar levels to healthy controls. As described in adults or adolescents, a blunted glucagon response and an increase in insulin have been observed in pre-pubescent children with T1DM (Adolfsson et al., 2012; Galassetti and Riddell, 2013). Furthermore, we observed that sub-maximal exercise was accompanied by a more pronounced GH peak in pre-pubescent children with T1DM than in the healthy children, consistent with previous data (Galassetti et al., 2006; Adolfsson et al., 2012). During exercise, GH increases endogenous glucose production and stimulates lipolysis in those considered healthy. However, fat oxidation is not increased during exercise (Møller and Jørgensen, 2009). This study’s findings for fat oxidation during exercise in children with T1DM were consistent with this observation.

This study has several inherent limitations that must be stated. Given that hormones (GH, cortisol, and glucagon) were not monitored continuously throughout the exercise test, in particular at the end of the exercise (T20), conclusions regarding the hormonal counter-regulation to exercise are limited. However, owing to the already high number of blood samples required by the protocol, and the previously described variations in the concentrations of hormones in response to exercise in non-diabetic adults and children, minimizing blood sample volume was decided. In future studies, the evaluation of body composition should be taken into account (especially lean body mass) along with measurements of blood lipid and lactate levels. Source of glucose oxidation rates evaluated by measurement of stable isotopes will to be considered. Various cardio-respiratory parameters (ventilatory threshold and heart rate variability threshold) need to be assessed in future exploration. The children with T1DM may have been less physically active in their everyday life. Assessment of their levels of physical activity *via* actimetry would be required to determine their spontaneous activity. Evaluation of adaptations to exercise in the same population but in a euglycemic state is also necessary. Finally, the conclusions from this study are only valid for moderate-intensity aerobic exercise. Obviously, the extrapolation to other physical activity modalities (e.g., aerobic or anaerobic and moderate or vigorous or high-intensity interval training) requires further investigation.

## Conclusion

The present study showed an impairment in physical fitness (with lower VO_2_ peak and a greater slope for heart rate vs. relative VO_2_ peak during exercise) and also in metabolic fitness (with lower MFO and lower substrate oxidation during submaximal exercise) in pre-pubescent children with T1DM. Exercise capacity is thus impaired early in the life-span of children with T1DM. Apart from deconditioning and poor glycemic control, this might reflect a decrease in oxidative metabolism in the skeletal muscle. Moderate-intensity exercise leads to a marked increase in muscle glucose uptake. Regular physical activity ≥3 times per week for ≥60 min each time should, therefore, be encouraged for all children with T1DM (Diabetes Canada Clinical Practice Guidelines Expert Committee et al., 2018) to reduce physical and metabolic impairments and improve quality of life and to decrease long-term complications.

## Data Availability Statement

The raw data supporting the conclusions of this article will be made available by the authors, without undue reservation.

## Ethics Statement

The studies involving human participants were reviewed and approved by Comité de Protection des Personnes (CPP) Est II. Written informed consent to participate in this study was provided by the participants’ legal guardian/next of kin.

## Author Contributions

SF, ER, EM, DT, and PD conceived the study. SF, ER, SE, EM, DT, and PD participated in the collection and statistical analysis of the data. BP performed the statistical analysis. SF, ER, GW, EM, DT, and PD participated in the interpretation of the results. SF and ER drafted the manuscript. GW, EM, DT, and PD critically reviewed the manuscript. All the authors read and approved the final manuscript. ER and PD are the guarantors of this work and, as such, had full access to all the data in the study and take responsibility for the integrity of the data and the accuracy of the data analysis.

### Conflict of Interest

The authors declare that the research was conducted in the absence of any commercial or financial relationships that could be construed as a potential conflict of interest.
